# Antiviral Activity of Quercetin Hydrate against Zika Virus

**DOI:** 10.3390/ijms24087504

**Published:** 2023-04-19

**Authors:** Marielena Vogel Saivish, Gabriela de Lima Menezes, Roosevelt Alves da Silva, Marina Alves Fontoura, Jacqueline Farinha Shimizu, Gislaine Celestino Dutra da Silva, Igor da Silva Teixeira, Natalia Franco Bueno Mistrão, Victor Miranda Hernandes, Paula Rahal, Lívia Sacchetto, Carolina Colombelli Pacca, Rafael Elias Marques, Maurício Lacerda Nogueira

**Affiliations:** 1 Laboratório de Pesquisas em Virologia, Departamento de Doenças Dermatológicas, Infecciosas e Parasitárias, Faculdade de Medicina de São José do Rio Preto, São José do Rio Preto 15090-000, SP, Brazil; 2 Brazilian Biosciences National Laboratory, Centro Nacional de Pesquisa em Energia e Materiais (CNPEM), Campinas 13083-100, SP, Brazil; 3 Departamento de Biofísica e Farmacologia, Universidade Federal do Rio Grande do Norte, Natal 59072-970, RN, Brazil; 4 Unidade Especial de Ciências Exatas, Universidade Federal de Jataí, Jataí 75801-615, GO, Brazil; 5 Laboratório de Estudos Genômicos, Departamento de Biologia, Instituto de Biociências, Letras e Ciências Exatas, Universidade Estadual Paulista, São José do Rio Preto 15054-000, SP, Brazil; 6 Departamento de Microbiologia, Faceres Medical School, São José do Rio Preto 15090-000, SP, Brazil; 7 Department of Pathology, The University of Texas Medical Branch, Galveston, TX 77555-0609, USA

**Keywords:** Zika virus, natural products, antiviral activity, inhibition, in silico analysis

## Abstract

Zika virus (ZIKV) has re-emerged in recent decades, leading to outbreaks of Zika fever in Africa, Asia, and Central and South America. Despite its drastic re-emergence and clinical impact, no vaccines or antiviral compounds are available to prevent or control ZIKV infection. This study evaluated the potential antiviral activity of quercetin hydrate against ZIKV infection and demonstrated that this substance inhibits virus particle production in A549 and Vero cells under different treatment conditions. In vitro antiviral activity was long-lasting (still observed 72 h post-infection), suggesting that quercetin hydrate affects multiple rounds of ZIKV replication. Molecular docking indicates that quercetin hydrate can efficiently interact with the specific allosteric binding site cavity of the NS2B-NS3 proteases and NS1-dimer. These results identify quercetin as a potential compound to combat ZIKV infection in vitro.

## 1. Introduction

Zika virus (ZIKV), a positive-sense single-stranded RNA virus, was first identified in Uganda in 1947 in monkeys and later in humans in 1952 [1,2]. Until the mid-2000s, rare sporadic cases of human infections were found across Africa and Asia, typically accompanied by mild illness [3]. However, after a ZIKV outbreak was recorded on the Island of Yap (Federated States of Micronesia) in 2007 [4], the virus spread to several countries with subsequent outbreaks [5,6]. In 2016, the World Health Organization declared that the association of Zika infection with clusters of microcephaly and other neurological disorders constituted a Public Health Emergency of International Concern [7]. Since that time, hundreds of thousands have been infected with the virus, and several regions across the globe remain at high risk for future outbreaks [8,9]. Brazil has suffered the most from ZIKV outbreaks since its emergence in 2015, with >3560 congenital ZIKV cases from 2015 to 2021 [10]. The search for effective antiviral agents against ZIKV from natural or synthetic sources is ongoing; several research groups have screened various United States Food and Drug Administration (FDA)-approved drugs and synthetic and natural compounds in ZIKV infection cell-based assays [11,12,13,14].

Quercetin (2-(3,4-dihydroxyphenyl)-3,5,7-trihydroxy-4H-chromen-4-one) is one of the most abundant representatives of the flavonoid subclass of flavonols, with documented multi-biological activities and extensive use in traditional medicine [15,16]. Dietary intake of flavonoids ranges from 5 to 100 mg/day (quercetin and its glycosides account for about 75%), primarily consumed as fruits, vegetables, and tea [17,18]. Quercetin supplementation offers several therapeutic properties, including antioxidant [16], anti-inflammatory, immunoprotective actions [19], anti-carcinogenic, and antidiabetic effects [20,21,22], and may prevent many chronic diseases [23], as well as inhibit lipid peroxidation, platelet aggregation, and capillary permeability, and stimulate mitochondrial biogenesis [24]. The antiviral effects of quercetin have been explored for several other viruses [25,26,27,28,29,30,31,32,33,34,35,36,37,38,39,40], and its structure is similar to quercetin hydrate.

Because of these effects, we investigated quercetin hydrate’s potential as a promising anti-ZIKV antiviral using in vitro tests and in silico approaches.

## 2. Results

### 2.1. Cytotoxicity of Quercetin Hydrate in Cell Culture

The MTT assay was used to determine the cytotoxicity of quercetin hydrate for Vero and A549 cells. Cell viability was well above 50% at the highest concentration (1000 µM), and we were consequently unable to determine a CC_50_ value (Supplementary Figure S1); cell viability corresponded to 81.2 ± 4.1% and 101.1 ± 12.9% for A549 and Vero cells, respectively, at 1000 µM. No cytotoxicity was observed in cells treated with 0.5% DMSO (final concentration of solvent used to dissolve compound, in cell culture media with cell viability of 79.8 ± 5.8% in A549 cells and 92.7 ± 10.1% in Vero cells).

### 2.2. Quercetin Hydrate Interferes with ZIKV Infection in a Dose-Dependent Manner

**Figure 1:**
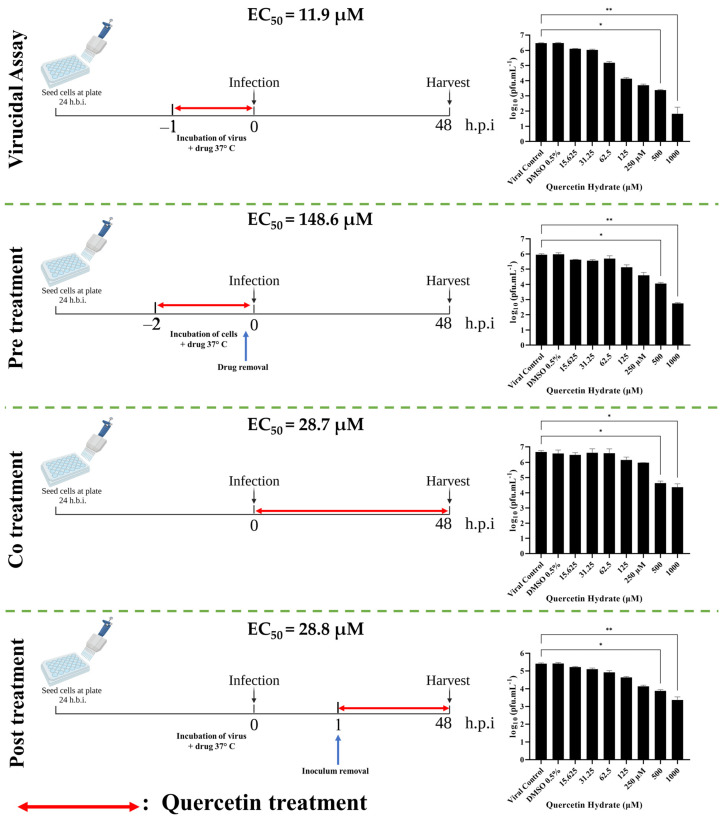
Quercetin hydrate causes dose-dependent inhibition of ZIKV yields. Vero cells were treated with DMSO solvent control or 1000–15.625 μM of quercetin hydrate in indicated conditions prior to infection with ZIKV at MOI = 0.1. The supernatant was titrated at 48 h post-infection. Mean values with standard deviation from three independent experiments, measured in triplicate, are shown. (*, *p* < 0.05/**, *p* < 0.01).

We initially tested the antiviral effect of quercetin hydrate on ZIKV in Vero cells since these cells are highly permissive to infection and are often used in related studies [41,42,43,44,45,46]. Vero cells were incubated with 1000–15.625 µM quercetin hydrate or the equivalent volume of DMSO and infected with ZIKV (MOI = 0.1) under different infection conditions for 48 hpi. Then virus yields were measured by viral titration (PFU/mL). The presence of DMSO did not affect the production of progeny infectious virus particles under any of the assay conditions (Figure 1).

In the virucidal assay to explore quercetin hydrate’s ability to interact with and functionally inhibit (neutralize) viral infectivity, EC_50_ was found to be 11.9 µM (95% CI 8.9–15.7 µM), and SI > 84.0 (calculated by dividing CC_50_ by EC_50_). The assay involving pre-treatment of the cells prior to infection found EC_50_ of 148.6 µM (95% CI 44.5–711.3 µM) and SI > 6.7. In the co-treatment assay, when the virus and compound were added to the cells simultaneously, EC50 was 28.7 µM (95% CI 9.9–88.1 µM) and SI > 34.8. Finally, in the post-treatment assay in cells 1 h after virus infection, EC_50_ was 28.8 µM (95% CI 22.4–37.1 µM) and SI > 34.7. These results indicate a significant dose-dependent decrease in the production of infectious ZIKV particles in the presence of increasing quercetin hydrate concentrations.

### 2.3. Quercetin Hydrate Reduces ZIKV Progeny Yield in Different Cell Lines

**Figure 2:**
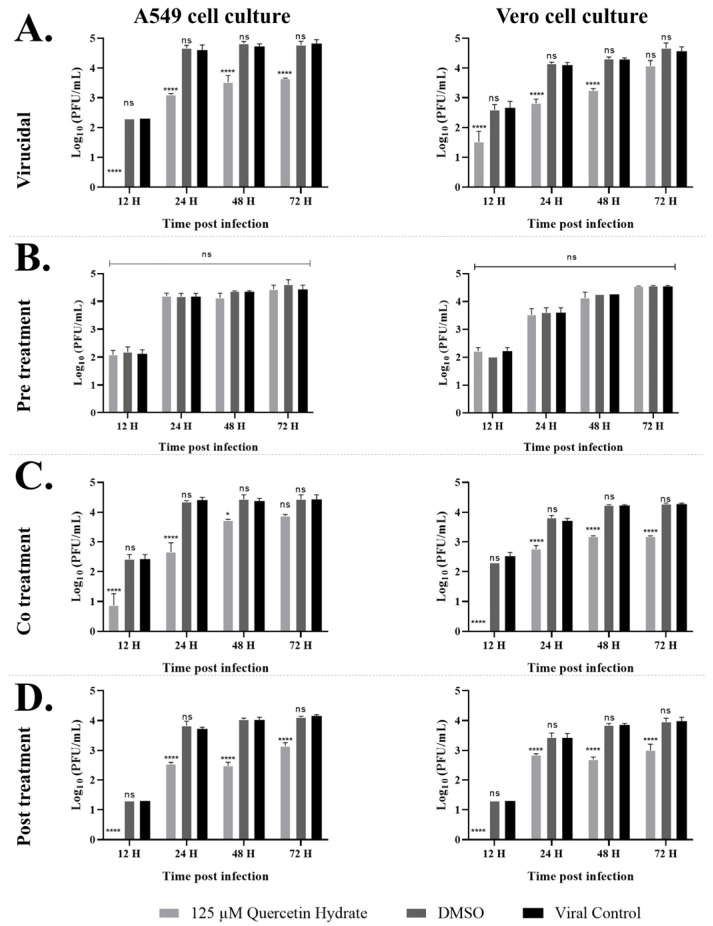
Growth curves of ZIKV (MOI = 0.1) in A549 or Vero cells in the presence of 0.5% DMSO or 125 µM quercetin hydrate, showing impaired ZIKV progeny production after treatment with quercetin hydrate when applied in the (**A**) virucidal assay, (**B**) pre-treatment assay, (**C**) co-treatment assay, and (**D**) post-treatment assay. (*, *p* < 0.05/****, *p* < 0.0001/ ns, not significant).

After observing the dose-dependent antiviral potential of quercetin hydrate in Vero cells, we performed kinetic infection in Vero or A549 cells to validate these findings in a cell line more relevant to human infection. The cells were pre-treated with 125 µM quercetin hydrate, DMSO, or untreated in the previously described conditions. Viral progeny production in the cell supernatants was quantified by plaque assay at the indicated post-infection times to observe multiple rounds of replication over 72 h. As shown in Figure 2, a time-dependent increase in viral titers was seen in DMSO-treated cells with comparable titers in the untreated controls. At the same time, quercetin hydrate caused a reduction in viral yields in three treatment conditions, decreasing the log10 titer of viral RNA copy number in cell content under virucidal, co-treatment, and post-treatment conditions (Supplementary Figure S2A,C,D).

To test whether the antiviral activity of quercetin hydrate towards ZIKV results from a direct virucidal effect of this compound on the viral particle, 125 µM quercetin hydrate was incubated with ZIKV (MOI = 0.1) at 37 °C for 2 h. This treated virus was then added to the cells, and the number of virus particles was determined via plaque assay at indicated times. At this concentration, quercetin hydrate had an antiviral effect in A549 and Vero cells (Figure 2A). Although the values for these time points corresponded to rounds of ZIKV replication, we observed reductions at up to 72 hpi for both cell lines tested. During the first 12 hpi, plaque formation was inhibited by 99.4 ± 0.1% and 92.7 ± 17.6% in A549 and Vero cells, respectively. Even at 72 hpi, the ZIKV titer was reduced from 4.8 log PFU (viral control) to 3.6 log PFU in A549 cells, a reduction of over 93%. In Vero cells, the ZIKV titer was reduced from 4.6 log PFU (viral control) to 4.0 log PFU, a more than 67% drop at 72 hpi. Average viral RNA titer decreases equivalent to 75.5%, 96.5%, 65.0%, and 42.9% were observed in the number of ZIKV RNA copies in the virucidal assay at 12, 24, 48, and 72 hpi, respectively, in A549 cells (Supplementary Figure S2A). In Vero cells, the average decrease in viral RNA was 32.2%, 94.1%, 57.7%, and 57.9% at 12, 24, 48, and 72 hpi, respectively (Supplementary Figure S2A).

Notably, previous incubation of quercetin hydrate with cells alone two hours before infection and removal of the compound prior to virus inoculation did not affect infectious titer compared to the DMSO control or viral control (Figure 2B). No reduction in the log10 titer of viral RNA copy number was observed in the pre-treatment assay (Supplementary Figure S2B); this indicates that the antiviral effect of quercetin hydrate is not the result of a preventive effect on the ZIKV particle at the concentration tested in the pre-treatment assay. However, this hypothesis cannot be ruled out since incubation for 2 h with the cells may not have been sufficient for cellular diffusion at an effective concentration of action. Another possibility is that the tested concentration of 125 µM may be insufficient to identify action in this specific assay condition since this concentration is below the EC_50_ value identified in Vero cells (Figure 1).

When the virus was mixed with quercetin hydrate and used for infection (in the co-treatment assay), a 97% reduction in virus titer was observed at 12 hpi in A549 cells and 99.6% in Vero cells (Figure 2C). Even at 72 hpi, the average ZIKV titer was reduced from 4.4 log PFU (viral control) to 3.8 log PFU in A549 cells (corresponding to 72% viral inhibition) and from 4.2 log PFU (viral control) to 3.1 log PFU in Vero cells (92% viral inhibition). Quantitative RT-PCR showed an average decrease of 43.5%, 64.4%, 27.9%, and 33.7% in ZIKV RNA copy number for the co-treatment assay in A549 cells at 12, 24, 48, and 72 hpi, respectively, compared to the untreated cells. In Vero cells, an average decrease of 41.7%, 43.2%, 65.0%, and 18.0% in ZIKV RNA copy number was observed at 12, 24, 48, and 72 hpi, respectively (Supplementary Figure S2C).

Treatment of the cells one hour after infection resulted in a 94% reduction in plaque-forming units at 12 hpi in both cell cultures (Figure 2D). At 72 hpi, the mean titer dropped from 4.2 to 3.1 log10 (90.3% reduction in PFU titer) in A549 cells and from 3.9 to 3.0 log10 in Vero cells (89.5% reduction in PFU titer). A reduction of approximately 68%, 39%, 73.5%, and 5.9% in average viral RNA titer was observed at 12, 24, 48, and 72 hpi, respectively, in A549 cells in the post-treatment assay (Supplementary Figure S2D). In Vero cells, an average decrease in the ZIKV RNA copy number of 35.4%, 33.9%, 64.4%, and 29.4% was observed at 12, 24, 48, and 72 hpi, respectively (Supplementary Figure S2D).

**Figure 3:**
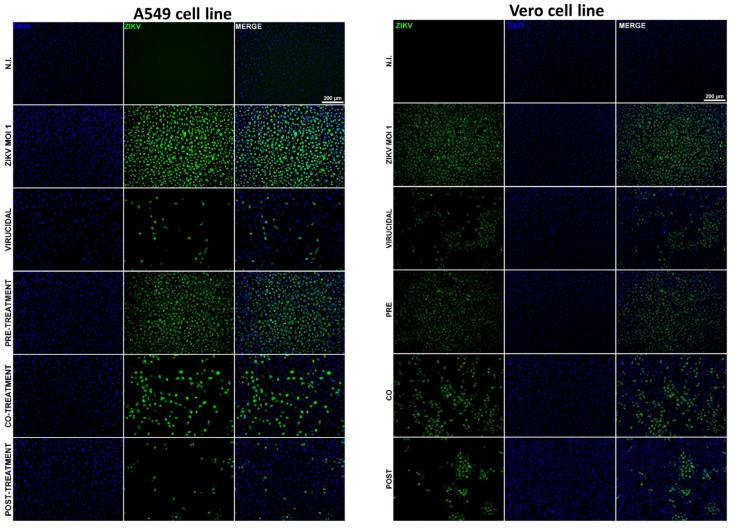
Evaluation of antiviral effect by indirect immunofluorescence assay (IFA). ZIKV was cultured at MOI = 1 in A549 or Vero cells with 125 μM quercetin hydrate. Non-infected cells and ZIKV-infected cells without compound were used as controls. After 12 h of incubation with the treatments, cells were fixed, and the expression of ZIKV E protein (in green) was detected with anti-E protein 4G2 mouse primary antibodies, followed by Alexa Fluor 488 mouse secondary antibody. Cell nuclei were stained with DAPI (in blue).

To confirm these results, we evaluated the presence of viral antigens in infected cells treated with quercetin hydrate and untreated controls using a qualitative immunofluorescence assay. A549 or Vero cells were subjected to the various treatment conditions described above with quercetin hydrate or DMSO and then infected with ZIKV (MOI = 1). After 12 h of incubation in each assay condition, the cells were fixed and stained with anti-Flavivirus envelope protein mouse primary 4G2 antibody, followed by Alexa Fluor 488 mouse secondary antibody. Cell nuclei were stained with DAPI (in blue). These assays revealed that quercetin hydrate treatment promoted a considerable decrease in the cells stained positive for viral antigens in the virucidal, co-treatment, and post-treatment assays (Figure 3); no reduction was observed in the pre-treatment assay. Together, these results indicate that quercetin hydrate affects ZIKV progeny yields but also suggest that this compound’s virucidal activity could directly affect the viral particle, or the molecule could affect even viral replication mechanisms.

### 2.4. ZIKV NS1 Structures

All Flaviviruses have a gene that produces non-structural protein 1 (NS1), which contains 352 amino acids; NS1 is a monomer and does not play a role in viral pathogenesis, but some researchers have stated that NS1 can be found in the host membrane (membrane-bounded NS1, mNS1) as a dimer configuration [47,48]. Inside the cell, mNS1 plays essential roles in RNA replication as well as viral particle production [49,50].

Each NS1 monomer has well-described conserved domains: the N-terminal ß-hairpin (residues 1–30), which contains a ß-roll subdomain (residues 1–25); the wing domain (residues 31–180), which contains a discontinuous connector subdomain (residues 31–37 and 152–180); and the ß-ladder domain (residues 181–352) [51] (Supplementary Figure S3).

**Figure 4:**
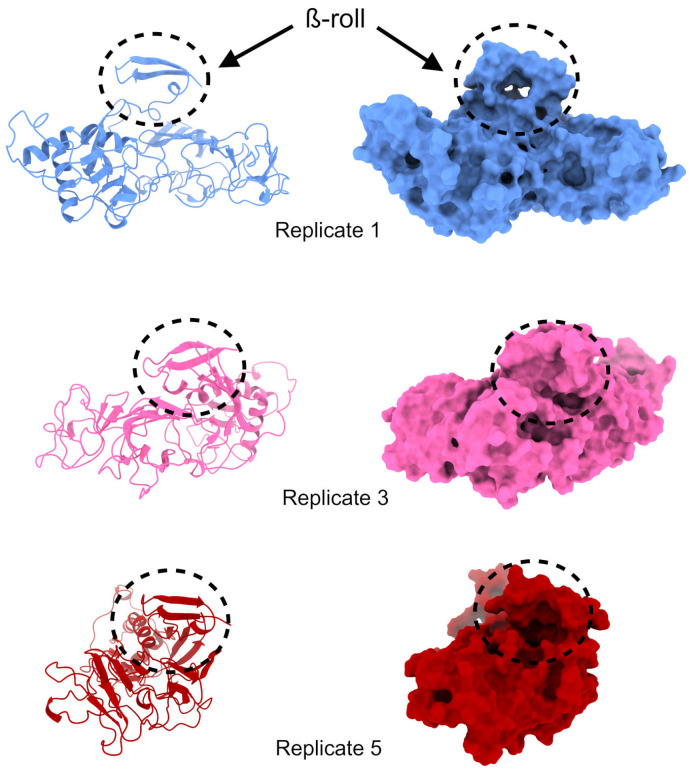
Structures selected for quercetin hydrate docking in the NS1 protein are represented as a ribbon and a surface for cavity visualization. The cavity comprising the ß-roll is demarcated in the black dashed lines.

For each MD replicate performed previously, 1500 NS1 structures were obtained. After clustering each replicate, 74, 48, 6, 21, and 24 clusters were observed for replicates 1, 2, 3, 4, and 5, respectively, using a 0.25 nm cut-off (see Figure S4). Visual inspection showed that the most present and stable cluster with a viable cavity in the ß-roll domain appeared in replicates 1, 3, and 5 (Figure 4). The depiction of replicate 2 shows that the ß-roll interacts with itself and with part of the NS1 loop, making it unfeasible for ligand interactions (Figure S5A). Similarly, in replicate 4, the ß-roll interacts with the ß-ladder domain (Figure S5B). This condition was previously hypothesized to make dimer formation unfeasible [52], and since the objective of this work is to avoid dimer formation, this configuration was not suitable for our purposes.

### 2.5. Exhaustive Docking

A summary of exhaustive docking is presented in Table 1. For NS2B-NS3, the values ranged from −7.3 kcal/mol to −7.4 kcal/mol. The AutoDock Vina (ADV) score for the crystallographic complex was −5.6 kcal/mol, which suggests quercetin has more affinity than MI-2227 for NS2B-NS3.

**Figure 5:**
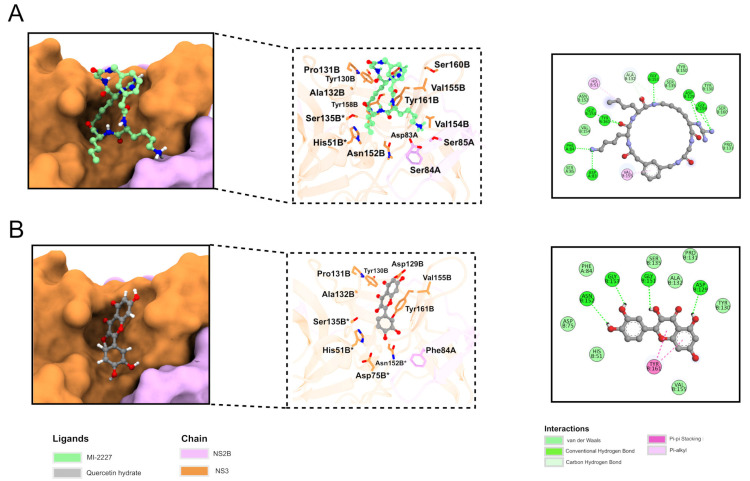
Binding mode of MI-2227 (**A**) and quercetin hydrate (**B**). Right panel: NS2B-NS3 is represented as a surface, while the ligands are shown as green (MI-2227) and gray (quercetin hydrate) stick structures. The NS2B and NS3 chains are presented as pink and orange, respectively. Center panel: protein is shown as a transparent ribbon, and residues interacting with the ligand are represented as stick structures. Left panel: 2D diagram of protein/ligand interaction. Residues that are part of the catalytic triad are marked with *.

In the 2D diagram of the interaction between NS2B-NS3 and ligand MI-2227, the interaction was seen between NS2B (final ‘A’) and especially the NS3 (final ‘B’) chain (Figure 5A). Hydrogen bond (Asp83A, Phe84A Asp129B, Glu151B, Gly153B, Gly159B, and Tyr161B), pi-alkyl (His51B and Val155B), van der Waals (Ser85A, Tyr130B, Pro131B, Ser135B, Tyr150B, Asn152B, Val154B, and Ser160B), and carbon-hydrogen (Ala132B) interactions can be observed. Two residues (His51B and Ser135B) are part of a catalytic triad.

The 2D diagram depicting the interaction between quercetin hydrate and NS2B-NS3 (Figure 5B) shows hydrogen bonds (Asp129B, Gly151B, Gly153B, Asn152B), pi stacking (Tyr161B), and van der Waals interactions (Phe84A, His51B, Asp75B, Tyr130B, Pro131B, Ala132B, Ser135B, Val155B). In this case, all residues of the catalytic triad (His51B, Asp75B, Ser135B) interact with the ligand. Twelve residues were seen to interact with both molecules (Phe84A, His51B, Asp129B, Tyr130B, Pro131B, Ala132B, Ser135B, Gly151B, Asn152B, Gly153B, Val155B, and Tyr161B); recent studies have shown the importance of His51B and Tyr161B in inhibitor binding, suggesting that quercetin binding could be suitable for NS2B-NS3 inhibition [53,54].

**Figure 6:**
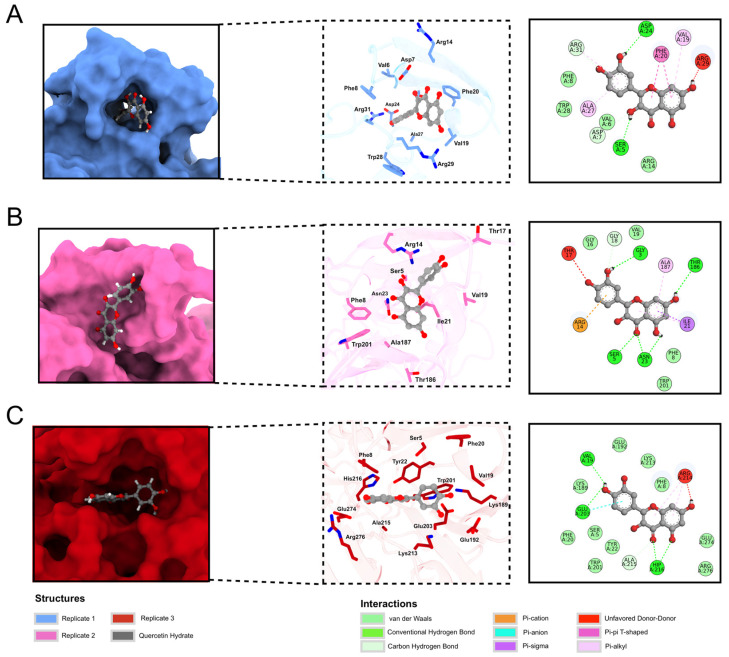
Best docking poses of quercetin hydrate in NS1 protein replicates. The ADV score for replicate 1 (**A**) was −6.985 kcal/mol, for replicate 3 (**B**) was −6.899 kcal/mol, and for replicate 5 (**C**) was −8.347 kcal/mol. The protein is represented as a surface (left panel) and stick structure (middle panel), and quercetin hydrate is represented as a gray ball and stick structure. Oxygen and nitrogen atoms are shown in blue and red, respectively.

For NS1 structures, exhaustive docking values ranged from −6.544 kcal/mol to −8.347 kcal/mol. The best (lowest) ADV score was seen in replicate 5, followed by replicates 1 and 3. Figure 6 illustrates the best binding mode for each replicate. Replicate 5 (Figure 6C) performed better than the others, with a cavity that allows quercetin hydrate to interact with more residues. Meanwhile, replicate 3 (Figure 6B) is more exposed to solvent, which reduces protein-ligand interaction. The quercetin hydrate in replicate 1 (Figure 6A) is partly inside and partly outside a cavity, which justifies its intermediate ADV score. Some residues can also be seen interacting with the ligand, with interactions in all replicates of NS1 (Ser5, Phe8, and Val19). A box plot of energy distribution can be seen in Supplementary Figure S6.

The ADV scores suggest that residues of the ß-ladder domain are more important for ligand binding and that ß-roll residues have a secondary (although still important) role in ß-roll stability since the best score was found for replicate 5 (Figure 6C), which demonstrates more interaction with residues from the ß-ladder domain. The dimeric NS1 interacting with envelope glycoproteins is essential for efficiently producing infectious viral particles and helps viral traffic inside cellular compartments in the host [50]. In this way, quercetin hydrate could inhibit the assembly and trafficking of viral particles, impeding them from being released in the extracellular medium to infect other cells. However, further studies are required to determine whether this ligand can stabilize the ß-roll domain.

## 3. Discussion

ZIKV is considered a public health threat, and while vector control is the most effective method to prevent the disease, the main concern in infection control is the lack of vaccines or antiviral therapies clinically approved. Drug discovery for this disease is challenging for scientists, so this study investigated quercetin hydrate, a candidate for new drugs derived from a natural source. This substance is abundant in plants and commonly consumed daily due to its presence in several foods, and it has been investigated to treat several diseases. Quercetin was determined to have the ability to inhibit the PD-1/PD-L1 interaction and act as an immune-enhancing cancer chemopreventive agent [55]. Quercetin demonstrates antibacterial activity against a wide range of bacterial strains, particularly those affecting the gastrointestinal, respiratory, urinary, and integumentary systems, with stronger antibacterial effects against Gram-negative than Gram-positive bacteria [56]. Still, quercetin and its derivatives have long been studied for their antiviral activity. In vivo, when combined with vitamin C, quercetin has helped prevent and treat patients with early respiratory infections, especially in patients with COVID-19 [57]. One clinical trial showed that patients with mild symptoms of COVID-19 who were treated with quercetin phytosome cleared the virus more quickly [58]; an in vivo study reported that oral quercetin provided some protection against lethal infection in Mengo virus-infected mice [57,58,59].

In vitro, quercetin and its derivatives have been shown to have antiviral activity against various viruses, including human herpesviruses [60], the H1N1 influenza A virus [61], the O’nyong’nyong virus [62], and some viruses in the family *Flaviviridae*. Quercetin was reported to have antiviral activity against the hepatitis C virus (HCV) through binding and inactivating viral NS3 protease [63]. The molecule was also reported to have antiviral activity against dengue virus 2 [64] as a non-competitive inhibitor of NS2B-NS3 protease [65]. Quercetin-3-β-O-D-glucoside was reported to have antiviral activity against the Zika virus, but no detailed mechanism of Q3G antiviral action was described [61]. We have identified the natural flavonoid quercetin hydrate as an antiviral compound against ZIKV. The strongest antiviral effects of this compound were observed when it was used in virucidal, co-treatment, and post-treatment assays, with EC_50_ values of 11.9, 28.7, and 28.8 µM, respectively. A higher EC_50_ value of 148 µM was seen when quercetin hydrate was used as a pre-treatment prior to infection, and no effect was observed in 125 µM concentration tests.

These results from the pre-treatment assay indicate that pre-incubation of the compound for a short period (2 h) followed by total removal of the drug may not be sufficient to generate a protective effect lasting long enough to influence later stages of the viral life cycle. Studies with a longer pre-incubation time for the compound may obtain different and perhaps more relevant results. Generally, EC_50_ values depend on the specific assay employed, and modifications (such as repeated administration of treatment) could result in slightly different EC_50_ values. We demonstrated that quercetin hydrate could control ZIKV replication for multiple rounds up to 72 hpi, with decreased progeny yields in virucidal, co-treatment, and post-treatment assays at 125 µM in A549 and Vero cells. Although our findings suggest that this compound may affect the immediate early phase (or potentially other phases) of virus replication, further research employing different assays is necessary to obtain a precise understanding of how quercetin hydrate acts as an antiviral.

In addition to the in vitro results, in silico information can help propose potential mechanisms for action that allow this compound to act directly on viral proteins; we used molecular docking to analyze the NS2B-NS3pro compound-protein and NS1-dimer. Favorable binding energies were observed between quercetin hydrate and the NS2B-NS3pro complex, suggesting that this molecule may be a promising inhibitor for NS2B-NS3pro. The NS2B-NS3 complex is multifunctional; the N-terminal region of NS3 and its cofactor NS2B fold into a protease responsible for viral polyprotein processing, and the C-terminal domain of NS3 possesses NTPase/RNA helicase activities and is involved in viral RNA replication and virus particle formation. Furthermore, the NS2BNS3 complex has also been shown to modulate viral pathogenesis and the host immune response [66]. An interesting study demonstrated that the citrus flavonoid naringenin was effective against distinct ZIKV lineages and may act as a non-competitive inhibitor of NS2B-NS3 protease [67]. Other molecules have also been reported as potential Zika virus NS2B-NS3 protease inhibitors [68,69,70]. As for the NS1-dimer, our findings suggest that quercetin hydrate could interact between binding points that could be key to NS1 dimerization. Interactions between the dimeric NS1 and envelope glycoproteins are crucial for the efficient production of infectious viral particles and assist in viral trafficking inside host cellular compartments [50]. These in silico results could explain the decrease in viral yields and genome copies in the ZIKV cultures treated with quercetin hydrate. However, we encourage further studies to be conducted to investigate the existence of these interactions. Future in vitro evaluations will help further elucidate these hypotheses. Finally, using computation tools to postulate potential mechanisms of action in antiviral compounds can help develop and rationally evaluate molecules with drug potential.

## 4. Materials and Methods

### 4.1. Cells, Virus, and Chemical

A549 (CCL-185—ATCC) and Vero (CCL-81 ATCC) cells were culture-grown in Minimal Essential Medium (MEM) (Gibco, Waltham, MA, USA) supplemented with 10% (*v*/*v*) heat-inactivated fetal bovine serum (FBS) (Gibco, Waltham, MA, USA), 100 U·mL^−1^ of penicillin, 0.1 mg·mL^−1^ of streptomycin, and 0.5 µg·mL^−1^ of amphotericin B (Gibco, Waltham, MA, USA) and incubated at 37 °C in a humidified atmosphere containing 5% CO_2_. The C6/36 cells were cultured in Leibovitz-15 medium (L-15) with 10% FBS at 28 °C. Zika virus (strain IEC 19—ICB/USP 2015) stocks were propagated in C6/36 cells and titrated in Vero cells to assess plaque formation, as described below in Item 2.5. Quercetin hydrate (2-(3,4-dihydroxyphenyl)-3,5,7-trihydroxy-4H-chromen-4-one hydrate) (Sigma-Aldrich, Saint Louis, MO, USA) was resuspended in dimethyl sulfoxide (DMSO) to create a 200 mM concentration stock solution and stored at −20 °C until use.

### 4.2. Cytotoxicity Analysis

Briefly, 5 × 10^4^ A549 or Vero cells grown in 96-well plates were treated with quercetin hydrate at concentrations ranging from 1000 µM to 31.25 µM for 48 h. Next, 1 mg/mL of 3-(4,5-dimethyl-2-thiazolyl)-2,5-diphenyltetrazolium bromide (MTT) (Sigma-Aldrich, Saint Louis, MI, USA) was added to the cells, and they were incubated for 1 h. Formazan crystals were dissolved in DMSO, and absorbance was determined at 550 nm using a Spectramax Plus microplate reader (Molecular Devices, Sunnyvale, CA, USA). Results are shown as the percentage of viable cells from the quercetin-hydrate-treated group relative to untreated control cells. For all assays, three independent experiments were performed in triplicate. From these data, the 50% cytotoxic concentration (CC_50_—cytotoxic concentration of the compound that reduced cell viability to 50%) was calculated from a dose-response curve in GraphPad Prism software (version 8.00) using four-parameter curve-fitting.

### 4.3. Definition of Viral Infection Assay Terms

Four different treatment conditions were used in time-of-drug experiments to explore which step(s) of the ZIKV replication cycle are blocked by quercetin hydrate. First, in a virucidal assay, the quercetin hydrate was added to the virus as a pre-treatment 1 h prior to inoculating of the treated virus into the cells to determine its virucidal or neutralizing activity. Next, a pre-treatment assay involved treating the cells with quercetin hydrate 2 h prior to viral inoculation. The third assay utilized co-treatment, with quercetin hydrate and virus simultaneously added to the cells, to determine the compound’s effect on virus entry, including virucidal (neutralizing) activity and blockade of viral attachment to and penetration into the cells. Finally, in a post-treatment assay, virus-infected cells were treated during the entire post-inoculation period to determine the antiviral effect of quercetin hydrate during post-entry steps such as genome translation and replication, virion assembly, and virion release from the cells. Viral infection experiments were performed in A549 or Vero cells seeded in 24-well or 48-well plates treated with or without quercetin hydrate, depending on the following tests described below.

### 4.4. Dose Response and Viral Growth Curve Assays

Assessment of the dose-response of quercetin hydrate on ZIKV was performed in 24-well plates (1 × 10^5^ cells per well); the cells were challenged with ZIKV (MOI = 0.1) and quercetin hydrate treatments ranging from 1000 µM to 15.625 µM. Cells treated with 0.5% DMSO and untreated cells were used as controls. The four treatment assays described in Section 2.3 were performed in Vero cells, and the supernatant virus plaque assay (described below in Section 2.5) was performed at 48 h post-infection (hpi). Three independent experiments were performed in triplicate, and data were analyzed by a four-parameter curve fitting from a dose-response curve using GraphPad Prism software (version 8.00) to calculate the half-maximal effective concentration (EC_50_—concentration of the compound that inhibited 50% of infection) with their confidence interval (CI). The selectivity index (SI) for the compound was determined as the ratio CC_50_:EC_50_.

To evaluate the viral growth curves in cells treated with quercetin hydrate and untreated controls, the assay was performed in 48-well plates (0.7 × 10^5^ cells per well). The A549 or Vero cells were challenged with ZIKV (MOI = 0.1) and treated with 125 µM of quercetin hydrate; cells treated with 0.5% DMSO and untreated cells were used as controls. The four treatment variations described in the time-of-drug assays in Section 2.3 were utilized, and the supernatant virus plaque assay (described below in Section 2.5) was performed at 12, 24, 48, and 72 h post-infection. Treated and untreated cells were compared using a one-way analysis of variance (ANOVA) followed by the Kruskal–Wallis post hoc test. All values were expressed as mean ± SD from at least three independent experiments, and all statistical tests were performed with GraphPad Prism software (version 8.0; GraphPad software, La Jolla, CA, USA).

### 4.5. Virus Plaque Assay

Briefly, Vero cells grown in a 24-well culture plate were infected with 0.1 mL of 10-fold dilutions of supernatants. Following 1 h of incubation at 37 °C, 0.5 mL of culture medium supplemented with 2% FBS and 1.5% carboxymethylcellulose sodium salt (Sigma-Aldrich, Saint-Quentin-Fallavier, France) was added, and the incubation was extended for three days at 37 °C. After removing the media, the cells were fixed with 10% formaldehyde and stained with 2% crystal violet diluted in 20% ethanol. Plaques were counted and expressed as plaque-forming units per milliliter (PFU·mL^−1^).

### 4.6. Immunofluorescence Assay

Vero or A549 cells were seeded onto 24-well plates (1 × 10^5^ per well) and incubated at 37 °C with 5% CO_2_ for 12 h. Cells were infected with ZIKV (MOI = 1), washed, and incubated with the compound for 24 h. Cells were washed and fixed in 4% paraformaldehyde, rewashed, and permeabilized with 0.05% Triton X-100 (JT Baker). Cells were blocked in 5% BSA (Sigma-Aldrich, Saint Louis, MI, USA) and incubated with an anti-flavivirus group antigen 4G2 antibody (Millipore, Burlington, MA, USA). Alexa Fluor 488 Rabbit Anti-mouse IgG (Abcam, Cambridge, MA, USA) and DAPI were used to stain viral proteins and cell nuclei, respectively.

### 4.7. RNA Extraction and RT-qPCR

To evaluate the viral growth curves in cells treated with quercetin hydrate and untreated controls, cells in 48-well plates (0.7 × 10^5^ cells per well) were challenged with ZIKV (MOI = 0.1) and treated with 125 µM of quercetin hydrate. The four treatment assays described in Section 2.3 were performed, and the cells were harvested at 12, 24, 48, and 72 hpi. Total RNA extraction was performed from cell culture content samples using a Bio Gene Viral DNA/RNA extraction kit (Bioclin, Belo Horizonte, MG, Brazil) according to the manufacturer’s instructions. The RNA obtained from the samples was used as a template in a one-step real-time polymerase chain reaction (RT-qPCR) using primers (forward primer All_S 5′ TACAACATGATGGGGAARAGAGARAA 3′ and reverse primer All_AS2 5′ GTGTCCCAGCCNGCKGTGTCATCWGC 3′) targeting the NS5 gene of the *Flavivirus* genus using the GoTaq 1-Step RT-qPCR System (Promega, Madison, WI, USA) [71]. Amplification was conducted in a QuantStudio 3 Real-Time PCR System (Thermo Fisher Scientific, Waltham, MA, USA) under the following conditions: reverse transcription at 45 °C for 15 min, denaturation at 95 °C for 10 min, and 40 cycles of 95 °C for 10 s, 60 °C for 30 s, and 72 °C for 30 s. A dissociation curve was subsequently conducted while gradually increasing the temperature from 60 °C to 95 °C. The results were analyzed in QuantStudio 3 software v1.5.1 (Thermo Fisher Scientific, Waltham, MA, USA). Treated cells and untreated controls were compared using two-way ANOVA followed by Tukey’s post hoc test. All values were expressed as mean ± SD from at least two independent experiments.

### 4.8. Obtention of Quercetin Hydrate Structure

The 3D structure of quercetin hydrate was retrieved from the PubChem website (https://pubchem.ncbi.nlm.nih.gov/compound/16212154 (accessed on 6 October 2022) in SDF file format. The structure was converted to PDBQT file format using Open Babel software [72], and the pH for hydrogens addition was settled to 7.4.

### 4.9. Obtention of ZIKV NS2B-NS3 Structures

The ZIKV NS2B-NS3 structure was downloaded from Protein Data Bank (PDB), and the criterion used was the resolution (PDB ID: 7ZYS, resolution: 1.26 Å). This structure already has an inhibitor binding (MI-2227) and was removed for docking purposes.

The ß-roll domain (residues 1–25) is the main interaction observed for dimer formation. Thus, the ZIKV NS1 structures were obtained after clustering focused on the ß-roll domain from MD performed previously in work executed by Menezes et al. [52,73,74,75]. In this work, five independent MD simulations were performed. The method of clustering based on the binding site is one of the most recommended, performing better than all-protein clusterization [76]. Hence, for clustering, we used the cut-off of 0.25 nm. We chose the most presented conformation of each replicate.

Since a pattern was not observed in the pocket formation for all replicates according to Menezes et al. (2022) [75] work, a visual inspection was performed to see if the ß-roll is in an open conformation that allows molecule binding. For NS1, a previous MD simulation is interesting since there is no NS1 monomeric structure in the PDB, and it can only be obtained from a dimeric structure. Hence, a conformational study of the monomer itself is necessary to obtain the dynamics of the ß-roll (which is highly flexible in monomeric configuration).

### 4.10. Exhaustive Docking

The docking site of NS2B-NS3 was defined the same as the molecule observed in the crystallographic structure. For each NS1 ZIKV structure, a grid box focused on the ß-roll region was designed to limit the searching space of the ligand. The quercetin hydrate was docked using the AutoDock Vina 1.2.2 (ADV) software [77]. The quercetin was docked 100 times in each ZIKV NS2B-NS3 and NS1 conformation to ensure the best result, resulting in 900 different binding modes. The best binding mode was analyzed using the 2D diagram from Discovery Studio Visualizer 2019 from Accelrys (https://discover.3ds.com/discovery-studio-visulizer-download (accessed on 13 October 2022). In addition, the ‘score_only’ function of ADV allows us to compare the score of MI-2227 with the quercetin hydrate binding to NS2B-NS3.

## 5. Conclusions

Quercetin hydrate has substantial antiviral activities against ZIKV in vitro. The observed antiviral effects were cell-type dependent, with slight differences between A549 and Vero cells. Further studies will better define the role of quercetin hydrate and its interaction with ZIKV. Our data expand the possibilities for future in vitro and in vivo testing of quercetin hydrate in animal models of flavivirus infection.

## Figures and Tables

**Table 1:** AutoDock Vina (ADV) docking results in kcal/mol.

Structure	Min.	1st Quartile	Median	Mean	3rd Quartile	Max.	SD ^†^
NS2B-NS3	−7.400	−7.300	−7.300	−7.319	−7.300	−7.300	0.0394277
NS1 rep. 1	−6.985	−6.775	−6.743	−6.735	−6.681	−6.544	0.0835779
NS1 rep. 3	−6.899	−6.656	−6.606	−6.634	−6.591	−6.568	0.0655191
NS1 rep. 5	−8.347	−8.011	−7.928	−7.974	−7.853	−7.830	0.1553613
7ZYS ^&^	−5.6723	-	-	-	-	-	-

SD ^†^ = standard deviation; ^&^ NS2B-NS3 crystal structure.

## Data Availability

Not applicable.

## Supplementary Materials

The following supporting information can be downloaded at: https://www.mdpi.com/article/10.3390/ijms24087504/s1.
