# Supplementary material for: Antiviral Activity of Quercetin Hydrate against Zika Virus
Source: Int J Mol Sci. 2023 Apr 19;24(8):7504. doi: 10.3390/ijms24087504 (PMC10144977; doi:10.3390/ijms24087504)
Supplement: Supplementary file 1 [file ijms-24-07504-s001.zip › ijms-2217122-Supplementary.pdf]

Supplementary Files

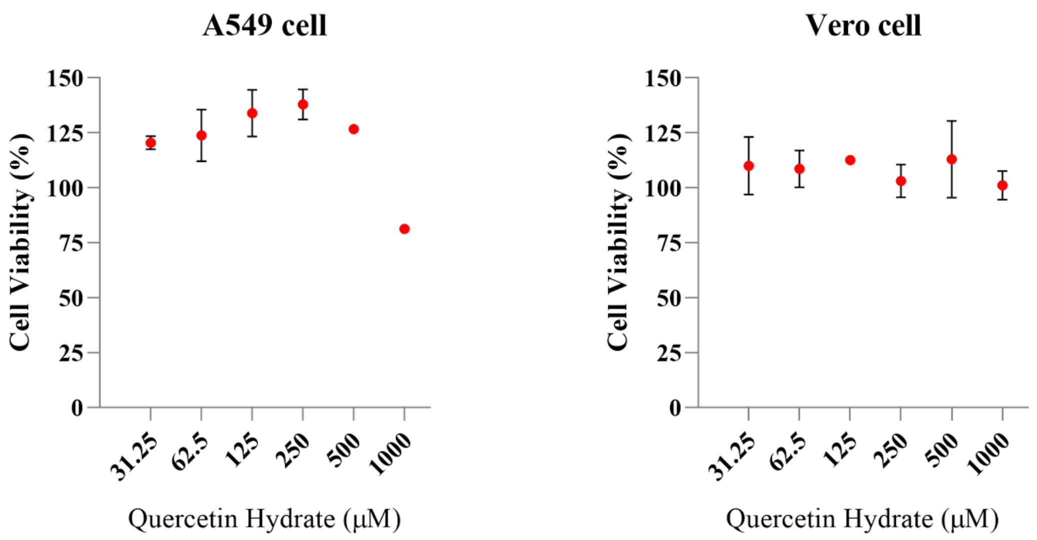

Figure S1 - Cytotoxicity of quercetin hydrate on A549 and Vero cells. After different concentrations of quercetin hydrate were applied to A549 and Vero cells for 48 h, cytotoxicity was evaluated using the MTT assay. All data are presented with error bars, and three independent experiments were performed.

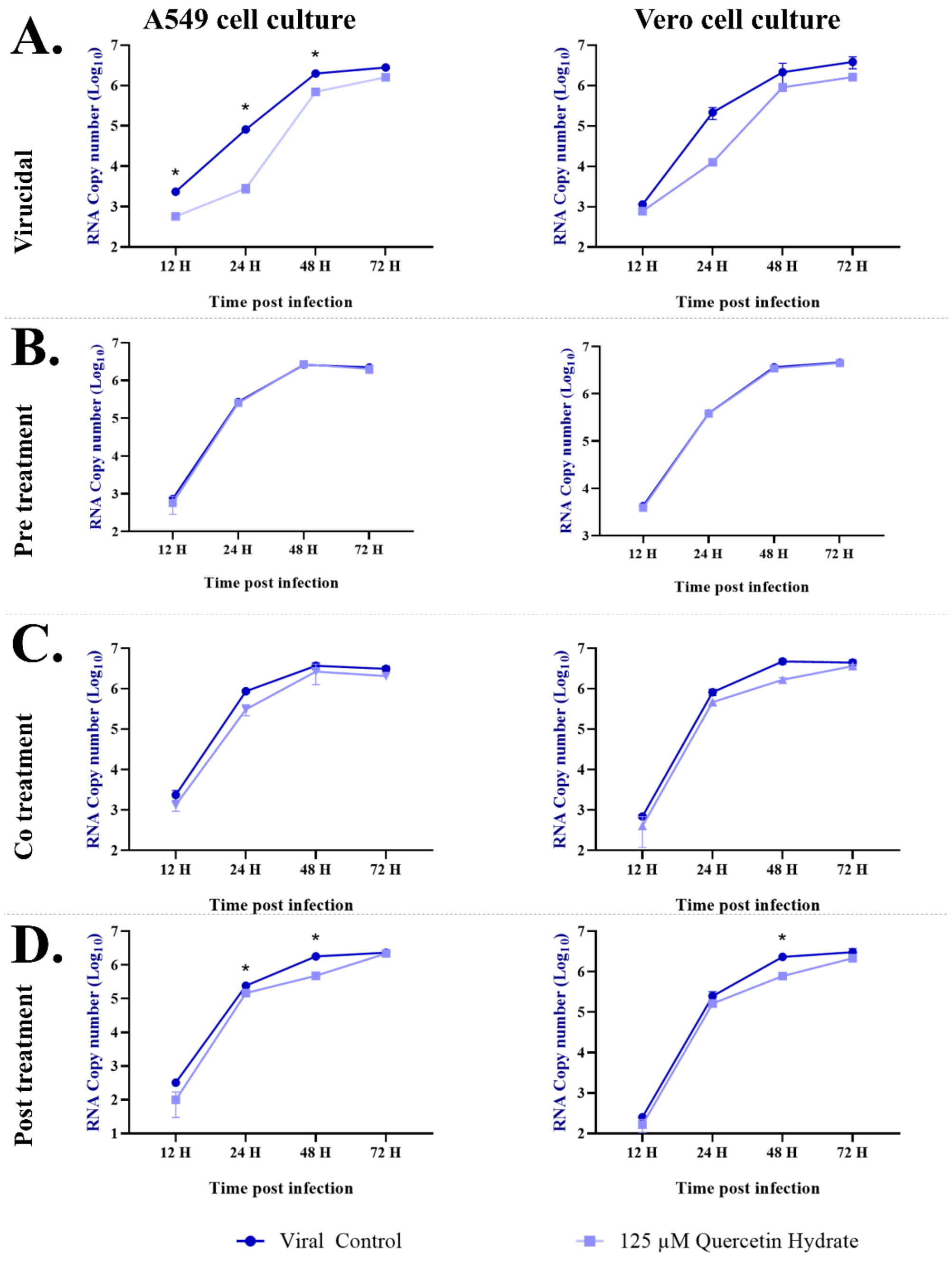

Figure S2. Effect of quercetin hydrate on ZIKV RNA levels, quantified by qRT-PCR after virucidal assay (A), pre-treatment (B), co-treatment (C), and post-treatment (D) conditions. Fold change was compared with the virus control (VC) and presented logarithmically. All findings are expressed as mean ± SD values from two experiments. Statistically significant differences between each compound and viral control are shown, with $P < 0.05$ considered significant (*$p \leq 0.05$).

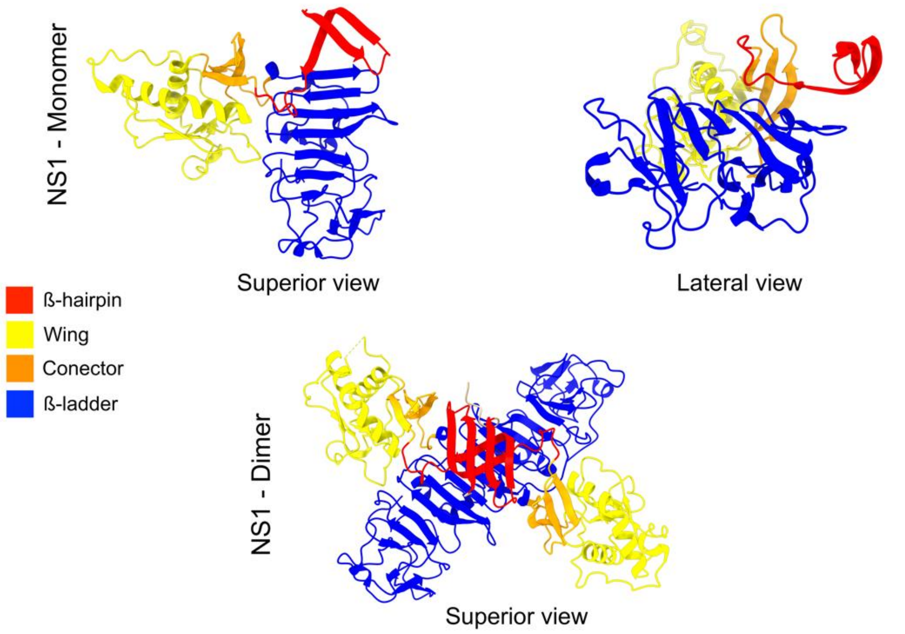

Figure S3 - Crystal structure of ZIKV NS1 (PDB ID: 5k6k) in ribbon representation. Top panel presents two different views of the monomer structure, while the bottom panel shows dimeric NS1. Note the interaction between the ß-hairpin domains to form the homodimer.

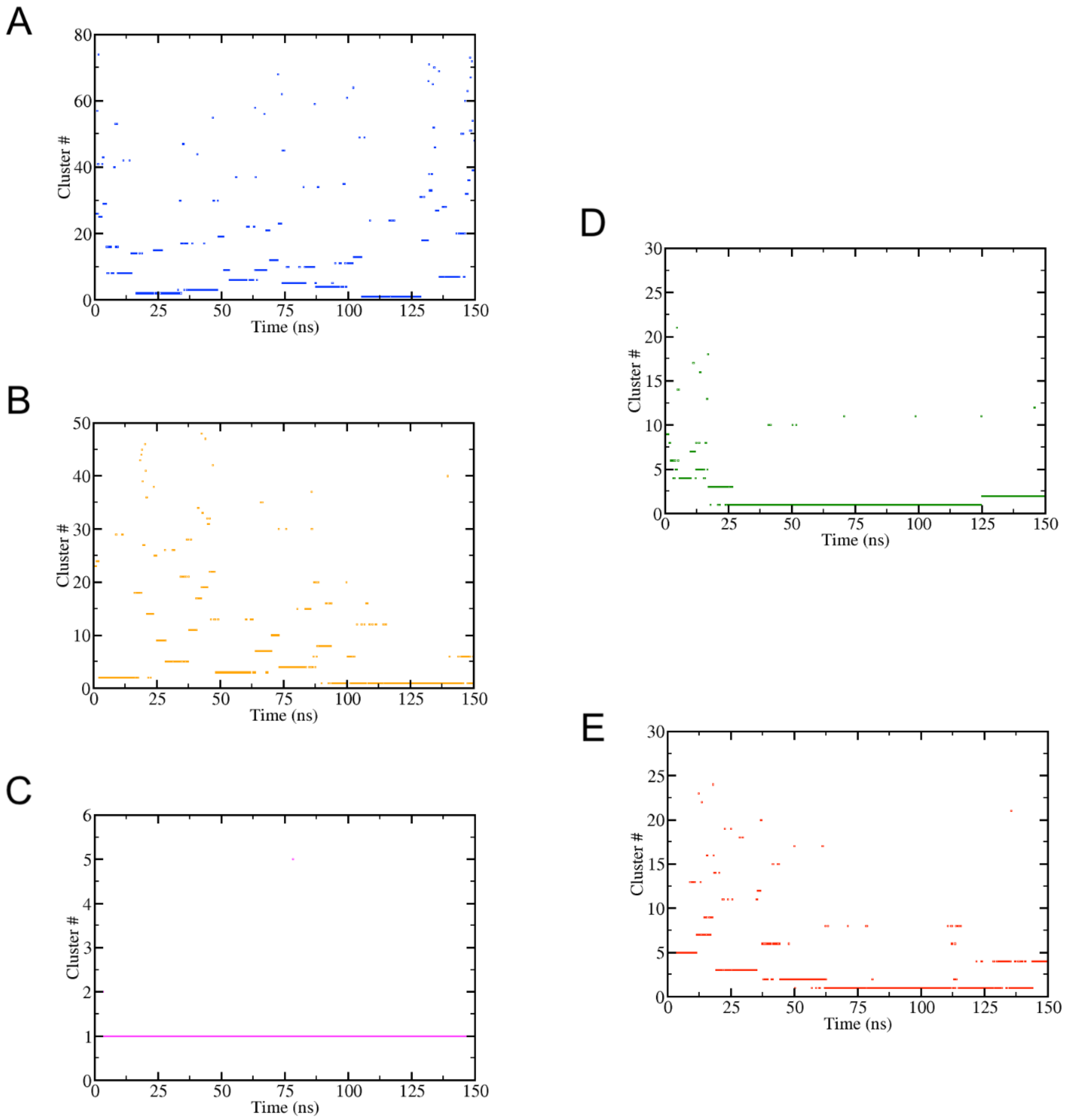

Figure S4 – Plot of cluster analysis based on 0.25 nm: (**A**) replicate 1, (**B**) replicate 2, (**C**) replicate 3, (**D**) replicate 4, and (**E**) replicate 5.

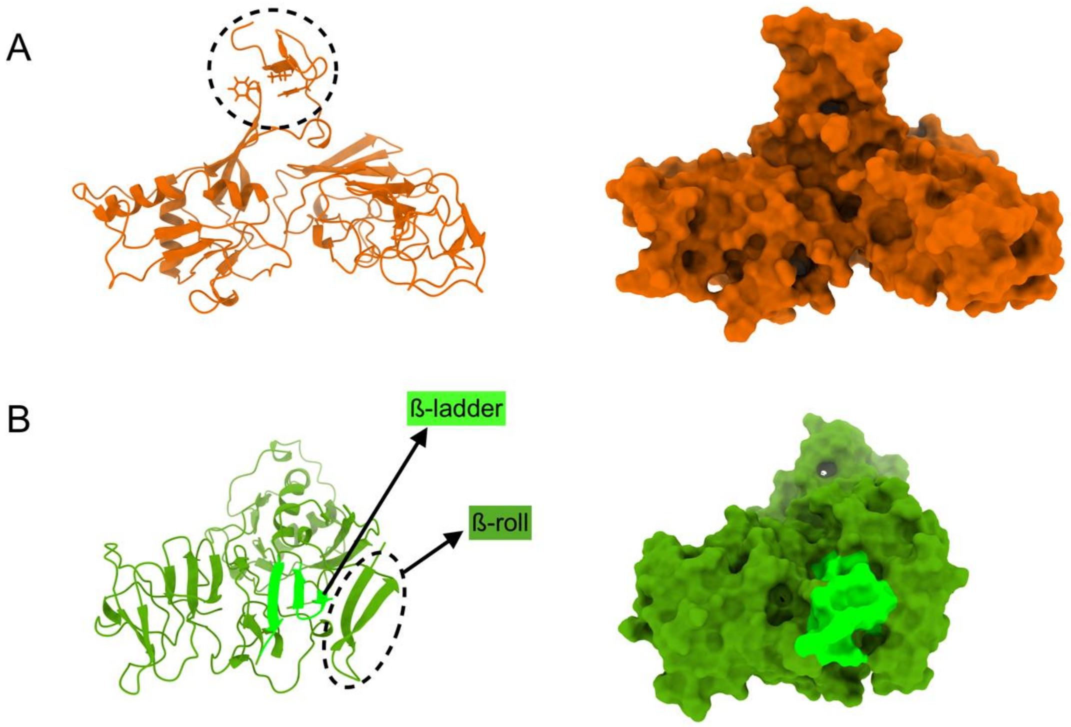

Figure S5 – Cluster 1 structure from replicate 2 (**A**) and replicate 4 (**B**).

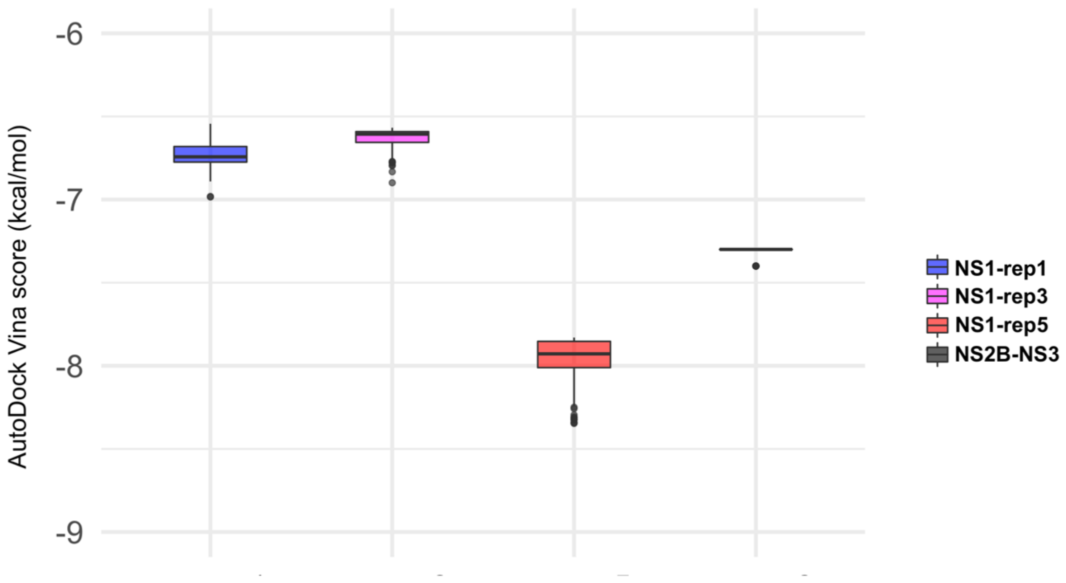

Figure S6 – Boxplot of ADV score distribution of 100 exhaustive docking.
